# Neurofeedback in Football: A Systematic Review of Cognitive, Technical, Physical and Psychological Outcomes

**DOI:** 10.3390/neurosci7030050

**Published:** 2026-04-23

**Authors:** Sílvio A. Carvalho, Pedro Bezerra, José E. Teixeira, Pedro Forte, Rui M. Silva, José M. Cancela-Carral

**Affiliations:** 1 Faculty of Educational Sciences and Sports Sciences, University of Vigo, 36005 Pontevedra, Spain; chemacc@uvigo.es; 2 SPRINT—Sport Physical Activity and Health Research & Innovation Center, 4900-347 Viana do Castelo, Portugal; pbezerra@esdl.ipvc.pt (P.B.); jeteixeira@ipca.pt (J.E.T.); ruimiguelfps@hotmail.com (R.M.S.); 3 Department of Sports, Higher Institute of Educational Sciences of the Douro, 4560-708 Penafiel, Portugal; 4 School of Sports and Leisure, Polytechnic Institute of Viana do Castelo, 4900-347 Viana do Castelo, Portugal; 5 Sports Science Department, Polytechnic of Cávado and Ave, 4750-810 Guimarães, Portugal; 6 CI-ISCE, ISCE Douro, 4560-708 Penafiel, Portugal; 7 Research Center for Active Living and Wellbeing (Livewell), Instituto Politécnico de Bragança, 5300-253 Bragança, Portugal; 8 Sports Science Department, Instituto Politécnico de Bragança, 5300-253 Bragança, Portugal

**Keywords:** EEG, cognition, psychophysiology, biofeedback, soccer

## Abstract

This systematic review synthesized the existing evidence on neurofeedback interventions applied to football players, aiming to clarify their effects on cognitive, technical–tactical, physical and psychological performance. Following Preferred Reporting Items for Systematic Reviews and Meta-Analyses (PRISMA) guidelines, four databases (PubMed, Web of Science, SCOPUS and SportsDiscus) were searched up to November 2025. Seven studies met the inclusion criteria, involving 133 players across youth, amateur, national and elite levels. Neurofeedback protocols primarily targeted alpha or sensorimotor rhythm (SMR) activity, and some were combined with heart rate variability (HRV) biofeedback. Across studies, neurofeedback may be associated with improvements in several cognitive outcomes, including improvements in working memory, visuospatial memory, task switching, mental rotation and decision-making. Limited evidence suggests potential improvements in technical skills (particularly shooting accuracy) and tactical decision-making. Some studies reported changes in physiological markers and stress-recovery capacity, although their interpretation remains uncertain. However, the evidence base remains constrained by small samples, heterogeneous protocols and limited use of randomized controlled designs. Overall, neurofeedback appears to be a potentially promising but still experimental tool to support cognitive and psychophysiological readiness in football, warranting more rigorous and standardized research to establish efficacy and optimal training parameters.

## 1. Introduction

Football is a multidisciplinary team sport that combines demanding physical actions with complex cognitive processes, requiring players to manage intermittent bursts of sprinting, rapid directional changes and periods of lower activity while maintaining quick and accurate decision-making under pressure [1]. Although physical load monitoring has advanced considerably, mental preparation strategies remain comparatively underexplored in football [2,3].

Neurofeedback has emerged as a promising approach to enhance cognitive and athletic performance by providing real-time information on brain activity and enabling individuals to adjust neural patterns to achieve desirable cognitive states [4]. Through operant conditioning, it can improve concentration, relaxation and sensorimotor control via training of specific EEG frequency bands [5], and initial applications in sports suggest meaningful benefits for cognitive regulation and stress resilience in competitive environments [6]. Evidence indicates that neurofeedback enhances cognitive skills such as working memory and attentional control, which are crucial for players operating in dynamic positional roles [7,8], with studies in football showing gains in working and visuospatial memory that support the attentional demands of defenders and attackers alike [9]. The Italian national team’s “Mind Room” during the 2006 World Cup exemplifies the practical application of neurofeedback and biofeedback to improve focus and emotional regulation in high-pressure contexts [10,11]. Research across sports further supports the safety and efficacy of EEG neurofeedback [12], and emerging multimodal frameworks highlight its potential to refine mental preparation tailored to the tactical and cognitive demands of different roles within a football team [13].

Although anecdotal reports highlight potential benefits of neurofeedback interventions in sport, including football, empirical evidence remains limited, with only a few studies suggesting improvements in reaction time and affective processes and very little research specifically targeting football players; for example, a behavioral case study by Diotaiuti et al. [14] did not address football directly but illustrated neurofeedback’s relevance for psychological practice, reinforcing the need for more focused investigations. Systematic reviews have largely overlooked football and other team sports, despite the crucial interaction between cognitive and physical performance, and while some literature explores EEG neurofeedback’s capacity to enhance cognitive functions, its application in football remains underexamined [15]. Much of the existing neurofeedback research is concentrated on clinical populations such as individuals with ADHD, leaving a gap in competitive sport contexts [16]. Recent reviews further emphasize the scarcity of studies addressing football-specific cognitive demands, suggesting opportunities to investigate how neurofeedback may support decision-making under pressure and sustained focus in football athletes [17]. Given the increasing interest among coaches and sport psychologists in mental training, a systematic synthesis of current evidence and innovative applications of neurofeedback is both timely and necessary to establish coherent methodologies tailored to football and to guide the development of effective cognitive training strategies capable of enhancing on-field performance. Therefore, the aim of this review was to systematically identify studies applying neurofeedback or integrated neurofeedback programs to football players.

## 2. Materials and Methods

### 2.1. Literature Search Strategy

The present review was conducted according to the Preferred Reporting Items for Systematic Reviews and Meta-Analyses (PRISMA) guidelines and the Population–Intervention–Comparator–Outcomes–Study design (PICOS) framework. The literature search strategy was registered on the International Platform of Registered Systematic Review and Meta-Analysis Protocols (INPLASY) with the number 202620042. Four electronic databases were systematically searched: PubMed/MEDLINE, Web of Science (all Core Collection citation indexes), SCOPUS, and SportsDiscus. Because the topic combines sport performance with neurophysiology, these databases cover both biomedical and sport-science literature. The search was designed to identify all peer-reviewed journal articles published until 15 November 2025 (final search in November 2025) involving neurofeedback interventions for football (soccer) players. No limits were applied for language, but only articles accessible in English were analyzed.

Search terms were structured around the PICOS model (Table 1). For the population, terms related to football (soccer) and futsal were used. The intervention terms targeted neurofeedback and related concepts, including electroencephalographic (EEG) neurofeedback, biofeedback, peak performance training and brain–computer interface (BCI). Because some programs combine EEG with autonomic biofeedback (e.g., HRV training) we included generic biofeedback terms to ensure that integrated neurofeedback methods were captured [18]. Comparator terms were not restricted because most neurofeedback studies use pre-post designs or sham/control groups. Outcome terms included cognitive function (e.g., attention, working memory, decision-making), psychomotor skill, physical performance and physiological responses. Boolean operators were used to combine terms, and the full search string for PubMed was: ((football OR soccer OR futsal) AND (neurofeedback OR “neurofeedback training” OR “EEG biofeedback” OR biofeedback OR “brain-computer interface”) AND (cognitive OR performance OR “working memory” OR attention OR decision OR psychomotor OR physical)).

Equivalent searches were adapted for the other databases. The search covered publications from database inception to 15 November 2025. References of all included articles and previous reviews were screened for additional eligible studies.

The database search was performed by one reviewer and checked by a second reviewer. Titles and abstracts were imported into a reference manager and duplicates were removed (Zotero, Corporation for Digital Scholarship. (2024). Zotero (Version 7.0) [Computer software]. https://www.zotero.org/ (accessed on 22 December 2025)). Two authors independently screened the titles and abstracts against the inclusion/exclusion criteria described below. Full-text articles were obtained for all studies meeting these criteria or when there was uncertainty. Disagreements were resolved through discussion or by consulting a third author.

### 2.2. Selection Criteria

The eligibility criteria were defined according to the PICOS framework: (1) Population: male and female football (soccer) or futsal players of any age and competitive level. Studies focusing on other team sports (e.g., rugby, Australian football) or on non-athlete populations were excluded. (2) Intervention: any form of neurofeedback training (EEG-based neurofeedback, brain–computer interface training, sensorimotor rhythm (SMR) protocols, alpha-band neurofeedback) delivered alone or in combination with other biofeedback modalities. We included studies employing integrated autonomic biofeedback programs if EEG neurofeedback formed part of the intervention. Studies that only assessed resting EEG without a feedback component were excluded. (3) Comparators: eligible studies included randomized controlled trials (RCT), quasi-experimental (pre-post) designs, case series and pilot studies with or without control/sham conditions. Cross-sectional studies without an intervention and purely descriptive neuroscience studies were excluded. (4) Outcomes: behavioral, cognitive, psychomotor, technical or physiological outcomes related to football performance. Examples include working memory, attention, decision-making, technical skills (e.g., shooting accuracy), physical tests (e.g., Yo-Yo Intermittent Recovery Test, YYIR), physiological metrics (e.g., EEG spectral power, peak alpha frequency), injury prevention and psychological states (e.g., resilience or flow). Studies that reported only biochemical markers or injury rehabilitation without neurofeedback were excluded. (5) Study design: original peer-reviewed research articles with full-text available. Reviews, commentaries, conference abstracts, theses, and editorials were excluded.

### 2.3. Quality Assessment

Methodological quality was evaluated using the modified Downs and Black Quality Index for cross-sectional studies and the Physiotherapy Evidence Database (PEDro) scale for intervention studies, following procedures adopted in previous systematic reviews [19]. The modified Downs and Black Index, applied to cross-sectional studies, consists of 14 items, with higher scores indicating greater methodological quality. For intervention studies, methodological quality was assessed using the PEDro scale, an 11-item instrument designed to evaluate RCT. Each item is scored dichotomously (0 or 1), and studies scoring six or higher are considered to be of high methodological quality [20]. Two reviewers independently rated each study. The inter-rater agreement (Cohen’s κ) was 0.90 (95% confidence interval 0.86–0.94); disagreements were resolved by consensus.

### 2.4. Study Coding and Data Extraction

For each included study, the following information was extracted: authors and year; country and competitive level; sample characteristics (sex, age, playing position, competitive level and sample size); neurofeedback protocol (targeted frequency band, electrode locations, number and duration of sessions, feedback modality and equipment); study design (randomized controlled trial, pre–post design or case series); comparator condition (control or sham group when available); outcome measures (cognitive, technical, physical, physiological and psychological variables); and main findings. Means ± standard deviations (SD), effect sizes, confidence intervals and *p*-values were recorded when provided.

## 3. Results

### 3.1. Search Results and Study Selection

**Figure 1:**
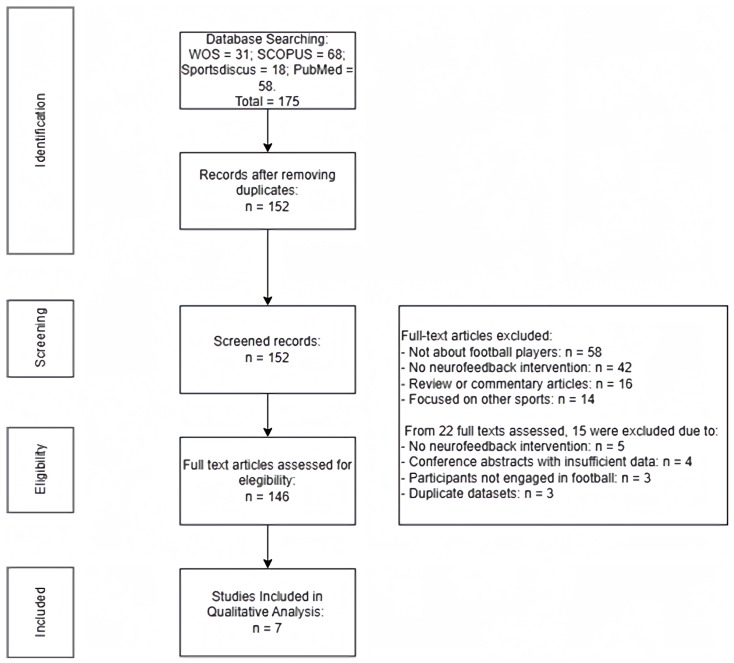
Studies selection flow diagram.

The search identified 175 records across the four databases (PubMed = 58, Web of Science = 31, SCOPUS = 68, SportsDiscus = 18). After removing 23 duplicates, 152 unique records remained. Title and abstract screening excluded 130 citations because they were not about football players (*n* = 58), did not include a neurofeedback intervention (*n* = 42), and were review/commentary articles (*n* = 16) or focused on other sports (*n* = 14). Full texts were obtained for 22 articles; however, 15 of these were excluded for the following reasons: lack of neurofeedback intervention (*n* = 5), results reported in conference abstracts without sufficient data (*n* = 4), participants not engaged in football (*n* = 3), and duplicate publication of the same dataset (*n* = 3). Ultimately, seven studies met the inclusion criteria and were included in the qualitative synthesis (Figure 1).

### 3.2. Participant Characteristics

The seven included studies involved a total of 133 football players. Sample sizes ranged from 5 to 48 participants. Five studies included male players only, one study included both males and females [21], and one study involved female players exclusively [22]. Participant ages varied from 14 to 32 years. Competitive levels ranged from youth academies and amateur clubs to national-level and elite professional teams. Only one study [23] reported anthropometric data (mean height 1.78 ± 0.06 m and body mass 74.5 ± 6.3 kg for 11 male professionals), making comprehensive synthesis of height and weight impossible.

### 3.3. Quality Assessment Results

For intervention studies, the PEDro score ranged between 6 (lowest quality) and 9 (highest quality) out of 11 points. For observational studies, Downs and Black scale was applied using scores ranging from 8 (lowest quality) to 12 (highest quality) out of a maximum of 14 possible points.

### 3.4. Data Organization and Synthesis of Findings

Table 2 summarizes key characteristics of the included studies. Because of heterogeneity in neurofeedback protocols, outcome measures and study designs, a quantitative meta-analysis was not possible. Instead, findings were synthesized narratively, grouped into four outcome domains: (i) cognitive performance, (ii) technical–tactical skills, (iii) physical and physiological responses, and (iv) psychological states.

### 3.5. Synthesis of Outcomes

Cognitive performance: Across studies, neurofeedback interventions targeted cognitive domains such as working memory, attention, and task switching. Although improvements were reported in several studies, variability in protocols, outcome measures, and study designs limits direct comparison and prevents firm conclusions regarding consistency of effects [21,22,24].

Technical–tactical skills: Only the SMR intervention study in female adolescents directly assessed technical outcomes. Carvalho et al. [22] found that increases in SMR activity following a six-week neurofeedback program were strongly correlated with improvements in shooting accuracy assessed through the Loughborough Soccer Shooting Test (LSST), as well as enhanced tactical decision-making measured through the FUT-SAT assessment. These findings suggest correlations between SMR-related changes and sport-specific performance measures, without establishing causality. Supporting these findings, a case-series study by Conde et al. [24] reported qualitative improvements in technical and tactical behaviors among youth football players following SMR neurofeedback training integrated into a sport psychology program. Although these results indicate potential benefits for football-specific skills, the limited number of studies and the predominance of non-controlled designs highlight the need for more rigorous research using validated sport-specific performance assessments [22,24]. These findings suggest a possible association between neurofeedback-related changes and sport-specific performance measures, although causal relationships cannot be established.

Physical and physiological responses: Four studies examined physical or physiological responses associated with neurofeedback interventions in football players. Carvalho et al. [22] reported that increases in SMR activity following a six-week neurofeedback program were positively associated with improvements in aerobic endurance performance, as reflected by higher scores on the YYIR. These findings suggest that neuromodulation targeting SMR frequencies may be associated with changes in physiological responses, although the underlying mechanisms and practical relevance remain unclear. Complementary evidence was reported by Rijken et al. [23], who implemented a multimodal program combining neurofeedback with heart rate variability (HRV) biofeedback and mental coaching in professional football players. Their results indicated significant increases in HRV indices, particularly the LF/HF ratio, alongside increases in EEG alpha power, reflecting enhanced autonomic regulation and improved psychophysiological readiness. Similarly, Rusciano et al. [18] reported improvements in physiological and stress-recovery profiles after an integrated neurofeedback and HRV biofeedback intervention in elite players; however, the contribution of neurofeedback-specific effects cannot be isolated from the multimodal design. In contrast, Belghadr et al. [4] did not observe significant changes in reaction time or other general physical performance indicators following a single alpha neurofeedback session, although a significant increase in peak alpha frequency was recorded. Taken together, these studies indicate changes in physiological markers, although their functional significance and direct relationship to performance remain unclear.

Psychological states: Several studies reported improvements in subjective outcomes such as sleep-related measures, flow and emotional stability. Boxtel et al. [21] observed increases in self-reported sleep duration and enhanced perceptions of flow and perceived control following group-based alpha neurofeedback training. Improvements in emotional stability and concentration were also reported in professional players participating in combined neurofeedback and HRV biofeedback programs [23]. Furthermore, Rusciano et al. [18] reported improvements in resilience and stress recovery, although these findings arose from a multimodal intervention and should not be attributed to neurofeedback alone. Early applied work, such as the “Mind Room” program described by Wilson et al. [11], also reported qualitative improvements in focus, relaxation and mental resilience among professional players. Together, these findings suggest potential effects on psychological well-being and emotional regulation, although much of the evidence is based on self-reported outcomes and limited-control designs.

## 4. Discussion

This review represents the first attempt to synthesize the effects of neurofeedback interventions in football. Despite the limited number of studies and their methodological heterogeneity, several noteworthy patterns emerged. The included interventions targeted alpha rhythms or SMR and were delivered either in isolation or as part of multimodal programs combining neurofeedback with HRV biofeedback or psychological skills training. Outcome measures spanned cognitive, technical–tactical, physical and psychological domains, and several studies reported changes across different outcome domains; however, these findings were heterogeneous in direction, magnitude, and methodological quality, limiting the identification of consistent patterns. The strength of evidence varies substantially across study designs. Only two studies employed randomized controlled designs, whereas the majority relied on uncontrolled or case-based approaches. This imbalance limits internal validity and restricts causal interpretation of the observed outcomes.

As for the cognitive performance, both RCT studies reported improvements in cognitive outcomes following alpha up-regulation. A single session of alpha neurofeedback increased peak alpha frequency and improved working and visuospatial memory [4], whereas repeated group sessions were associated with better task switching and mental rotation [21]. SMR training correlated with improved decision-making and tactical processing in female youth players [22]. Randomized trials showed that a single alpha-training session increased peak alpha frequency and improved working and visuospatial memory [6], while repeated group sessions were linked to gains in cognitive flexibility, including task switching and mental rotation [10]. SMR training has also been associated with better decision-making and tactical processing in female youth players [12], aligning with broader findings that modulation of alpha and SMR frequencies can improve attention, working memory, and cognitive flexibility [25]. Despite these promising outcomes, variability in neurofeedback protocols (such as session duration, feedback modalities, and reinforcement thresholds) limits comparability across studies and complicates general conclusions about efficacy [26], but may reflect a potential role of neurofeedback in neural activity modulation, although alternative explanations such as task familiarization or expectancy effects cannot be excluded [27]. However, the included studies did not directly manipulate arousal levels or experimentally examine arousal–performance relationships, and interpretations should therefore remain limited to the neural and behavioral outcomes reported in each study. Moreover, the interpretation of these findings requires caution. The available studies are characterized by small samples, heterogeneous protocols and limited use of adequately controlled designs. In addition, most cognitive outcomes were assessed using laboratory-based tasks rather than ecologically valid football-specific situations. As a result, it remains unclear as to what extent observed improvements reflect neurofeedback-specific effects, general task familiarization, expectancy effects, or meaningful transfer to real football performance.

Evidence regarding technical–tactical performance remains limited and methodologically constrained. Only one study quantitatively assessed sport-specific technical outcomes, reporting strong correlations between SMR activity and shooting accuracy and tactical decision-making. However, these findings originate from a small, uncontrolled sample, which precludes causal inference. Complementary qualitative evidence from a case-series design suggests potential improvements in technical and tactical behaviors, although these observations lack standardized measurement and control conditions. In that study, increases in SMR power strongly correlated with better shooting accuracy and tactical decisions [22]. Qualitative reports from a case series suggested improvements in technical and tactical behaviors [24]. Research examining neurofeedback’s impact on athletes’ technical and tactical skills suggests promising outcomes, with one study reporting qualitative improvements in peripheral visual attentional performance in professional football players after neurofeedback training, indicating potential benefits for tactical decision-making [28]. Nonetheless, the current body of evidence is limited, emphasizing the need for rigorous, well-controlled studies using validated sport-specific performance tests to determine whether neural improvements genuinely translate into enhanced technical skills and decision-making under competitive conditions [29]. Again, the variability in protocols (such as differences in session duration, feedback modalities, and reinforcement strategies) further complicates cross-study comparisons and hinders firm conclusions regarding neurofeedback’s effectiveness [15], making the standardization of methods an essential step for advancing knowledge in this field. Evidence regarding technical–tactical performance remains limited. Only one small uncontrolled study directly assessed football-specific technical outcomes quantitatively, while another provided only qualitative case-based observations. Although these results are of interest, they do not establish that neurofeedback causally improves sport-specific execution or decision-making. A key unresolved issue is whether neural or cognitive changes observed in training settings translate into meaningful improvements in match-relevant performance.

With respect to physical and physiological outcomes, the current evidence is limited and methodologically heterogeneous. One small uncontrolled study reported associations between SMR changes and endurance performance [22], whereas multimodal interventions combining neurofeedback with HRV biofeedback and mental coaching reported changes in stress-recovery profiles and HRV-related indices [18,23]. These findings should be interpreted cautiously. In particular, the LF/HF ratio is not an unequivocal marker of sympathovagal balance, and multimodal designs do not allow the specific contribution of neurofeedback to be isolated. No robust evidence currently demonstrates direct improvements in core football-relevant physical capacities such as sprinting, agility or neuromuscular performance.

Finally, as for the psychological states, several interventions produced improvements in subjective well-being. Group alpha neurofeedback increased sleep duration and feelings of flow [21] and is consistent with research linking better sleep quality to improved athletic outcomes [30], including performance [21] and linked to qualitative improvements in focus, relaxation and psychological readiness [11]. Additionally, combined neurofeedback and HRV training enhanced emotional stability, concentration and resilience [18,23]. Combined neurofeedback and heart rate variability training has also been associated with greater emotional stability, concentration, and resilience [30], stress and arousal regulation [31] as well as cognitive control [32]. Psychological outcomes also appear potentially responsive to these interventions, particularly in relation to sleep-related measures, emotional stability, concentration and flow. Nevertheless, these findings are based predominantly on subjective self-report outcomes and on designs with limited control over expectancy and placebo effects. For that reason, the available evidence supports only cautious interpretation, and it remains uncertain whether the observed benefits are attributable specifically to neurofeedback, to broader psychological support, or to non-specific effects of structured intervention.

Rather than indicating consistent effects across domains, the current evidence reflects a fragmented and heterogeneous research landscape. Variability in study design, intervention protocols, and outcome measures prevents the identification of clear patterns. As such, observed findings should be interpreted as preliminary signals rather than convergent evidence of efficacy.

### Future Research, Practical Applications and Study Limitations

Sample sizes ranged from 5 to 48 participants, and only two studies employed randomization or a control group. However, the heterogeneity of neurofeedback protocols, small sample sizes and the limited number of randomized controlled trials highlight the need for more rigorous and standardized research to clarify the magnitude and mechanisms of these effects. Consequently, the interpretation of the results should consider the statistical power reported in each study. The study findings should be interpreted cautiously, as many of the included studies involved small samples, uncontrolled pre–post designs or exploratory pilot approaches, which may increase susceptibility to reporting bias and expectancy effects. Blinding and allocation procedures were rarely described, and follow-up assessments were short or absent. Neurofeedback protocols varied widely, making it difficult to identify optimal training parameters. Furthermore, most studies focused on male players; female participants were represented in two studies, although the overall evidence remains predominantly based on male samples, and none included goalkeepers or futsal players. The generalizability of results for different ages, competitive levels and positions is therefore limited. Finally, a key limitation of the current evidence base is the difficulty in separating neurofeedback-specific effects from non-specific effects such as placebo responses, participant expectations, increased researcher attention, repeated testing and general cognitive engagement. This issue is especially relevant because sham-controlled conditions were rare and blinding procedures were often absent or insufficiently described. Consequently, the overall strength of evidence should be considered preliminary rather than confirmatory. Across studies, common methodological limitations included small sample sizes, absence of blinding procedures, limited use of sham-control conditions, and short or absent follow-up periods, indicating an overall elevated risk of bias. Additionally, inconsistent reporting of effect sizes across studies limits the ability to compare intervention magnitude and assess practical relevance.

Overall, the available evidence suggests that neurofeedback interventions may be associated with changes across multiple domains relevant to football performance. Cognitive outcomes appear to be the most frequently reported, whereas technical–tactical findings remain sparse and preliminary. Physiological responses mainly reflect improvements in autonomic regulation and recovery capacity rather than direct changes in physical performance variables. Psychological findings, including emotional stability, concentration and flow-related outcomes, are encouraging but remain limited by subjective measurement and low-control designs. Future research should address these limitations by conducting adequately powered randomized controlled trials with standardized protocols, appropriate control conditions (e.g., sham feedback), and longer follow-ups. Researchers should report individual responder patterns to explore whether factors such as baseline EEG characteristics, playing position or sex influence neurofeedback efficacy. Integrating objective performance tests and ecologically valid assessments (e.g., virtual reality decision-making tasks) will help determine whether neural changes translate to game-relevant improvements. The integration with artificial intelligence-based technology should also be tested [33,34]. Finally, comparative studies should investigate whether alpha, SMR or beta protocols yield differential benefits, and whether combining neurofeedback with HRV or mindfulness training produces additive effects.

## 5. Conclusions

This systematic review provides an initial overview of neurofeedback interventions in football. The limited and heterogeneous body of evidence indicates preliminary and inconsistent findings regarding neurofeedback interventions targeting alpha or SMR bands; however, evidence for transfer to on-field technical performance remains limited and preliminary. Programs combining neurofeedback with HRV biofeedback have been associated with reported improvements in physiological and psychological outcomes, although the specific contribution of neurofeedback cannot be isolated in such multimodal interventions. However, the heterogeneity of protocols and the predominance of small, uncontrolled studies preclude firm conclusions. More rigorous research is needed to establish efficacy, determine optimal training parameters and elucidate the mechanisms underpinning performance gains. Given the methodological limitations, small sample sizes, and heterogeneity of protocols, current evidence should be interpreted as exploratory rather than confirmatory.

## Figures and Tables

**Table 1:** Search terms used for the screening procedures.

Search Term	Keywords (Truncated Examples)
Football (population)	“football” OR “soccer” OR “futsal” OR “association football”
Neurofeedback intervention	“neurofeedback” OR “neurofeedback training” OR “EEG biofeedback” OR “brain-computer interface” OR “biofeedback”
Outcome/performance	“cognitive performance” OR “attention” OR “working memory” OR “decision making” OR “technical skill” OR “physical performance”
Boolean search phrase	population AND (intervention AND outcome)

**Table 2:** Characteristics of studies included in the systematic review on neurofeedback and football.

Study (Year)	Sample & Level	Neurofeedback Protocol	Design/Comparator	Outcomes	Main Findings	Quality
Boxtel et al. [21]	41 national-level players (15 male, 26 females; mean age ≈ 19 yr)	Music-based neurofeedback targeting alpha (8–12 Hz). Players trained in groups up to 13; eight 10 min blocks alternating neurofeedback and cognitive tasks.	Crossover design; pre/post measures of cognitive tasks.	Task switching, mental rotation; EEG alpha power; self-reported sleep and flow.	Neurofeedback increased alpha power by 34% and decreased beta activity; improved task switching and mental rotation performance; players reported longer sleep duration and greater feelings of control and flow.	9
Belghadr et al. [4]	48 male amateur players aged 18–28 yr (24 NFT, 24 sham)	Single 25 min session of alpha neurofeedback at Pz; reward threshold adjusted to increase peak alpha frequency (PAF).	RCT, sham-controlled; players stratified by playing position (defenders vs. attackers).	Working memory (NCR-L1, NCR-L2), visuospatial memory (FRS, BRS), reaction time and PAF.	NFT group improved NCR-L1, NCR-L2 and FRS scores (*p* < 0.05) with defenders showing larger gains than attackers; PAF increased from 9.72 Hz to 10.28 Hz (*p* = 0.046).	12
Carvalho et al. [22]	8 female youth players (14–18 yr) from a Portuguese club	Six-week SMR (12–15 Hz) neurofeedback program using a virtual football scenario; three 30 min sessions per week.	Pre–post design; no control group.	Anthropometrics; EEG; technical (LSST), physical (YYIR), tactical (FUT-SAT) and decision-making assessments.	Correlations between changes in neurofeedback outcomes and performance metrics ranged from 0.504 to 0.998 with *p*-values 0.010–<0.001; greater SMR increases were associated with better shooting accuracy, aerobic endurance and tactical decision-making.	9
Conde et al. [24]	5 male youth players; Brazilian Sport Club	Four-week SMR training at Cz (12–15 Hz); weekly sessions with audio-visual feedback; training integrated into sport psychology service.	Case series (pre–post); no control.	Behavioral complaints reported by athletes, parents, coaches and psychologists (technical, tactical, physical, psychological).	Four of five athletes were considered remitted from initial behavioral complaints; improvements varied individually, suggesting SMR neurofeedback may enhance functional behaviors in developing footballers.	6
Rijken et al. [23]	11 professional male players (mean age ≈ 22 yr) from a Dutch club	Mental coaching combined with daily HRV biofeedback and alpha-frequency (8–12 Hz) neurofeedback; 10 sessions.	Pre–post design with 5-week follow-up; no control.	Psychological scales (SIM-60 emotional stability and concentration), HRV (LF/HF ratio), EEG alpha power.	Significant improvements in emotional stability and concentration; LF/HF ratio increased from 2.53 to 7.52 (*p* = 0.02) and alpha power at C3, C4, Oz, P3, P4 increased (*p* = 0.02–0.03). Effects persisted at follow-up.	7
Rusciano et al. [18]	20 elite Serie-A players; Italy	“Neuroplus” integrated autonomic biofeedback (HRV and breathing) and neurofeedback training; 10 sessions over 5 weeks.	RCT (Neuroplus vs. control).	Psychophysiological stress profile, visual selective attention, injury prevention metrics.	Neuroplus group showed significant improvements in physiological adaptability, recovery from stress, visual selective attention and injury prevention compared to control.	11
Wilson et al. [11]	Single professional club (AC Milan)	“Mind Room” program combining neurofeedback, HRV biofeedback and psychological skills training; long-term implementation.	Case report.	Players’ performance and psychological state.	Described improvements in focus, relaxation and resilience among players; data insufficient for quantitative analysis.	6

Abbreviations: BRS—backward recall score; EEG—electroencephalography; FRS—forward recall score; FUT-SAT—System of Tactical Assessment in Soccer; HRV—heart rate variability; LF/HF—low-frequency to high-frequency ratio; LSST—Loughborough Soccer Shooting Test; NCR-L1—number cancelation test level 1; NCR-L2—number cancelation test level 2; NFT—neurofeedback training; PAF—peak alpha frequency; RCT—randomized controlled trial; SIM-60—Sport Imagery Measure-60; SMR—sensorimotor rhythm; YYIR—Yo-Yo Intermittent Recovery Test.

## Data Availability

The data supporting the findings of this study are not publicly available due to privacy or ethical restrictions but can be provided by the corresponding author upon reasonable request.
